# Left Ventricular Transmural Gradient in Mitochondrial Respiration Is Associated with Increased Sub-Endocardium Nitric Oxide and Reactive Oxygen Species Productions

**DOI:** 10.3389/fphys.2016.00331

**Published:** 2016-08-17

**Authors:** Michel Kindo, Sébastien Gerelli, Jamal Bouitbir, Tam Hoang Minh, Anne-Laure Charles, Jean-Philippe Mazzucotelli, Joffrey Zoll, François Piquard, Bernard Geny

**Affiliations:** ^1^Equipe d'Accueil 3072, Faculté de Médecine, Institut de Physiologie, Université de StrasbourgStrasbourg, France; ^2^Service de Chirurgie Cardiovasculaire, Pôle d'activité Médico-Chirurgicale Cardiovasculaire, Nouvel Hôpital Civil, CHRU de StrasbourgStrasbourg, France; ^3^Service de Physiologie et d'Explorations Fonctionnelles, Pôle de Pathologie Thoracique, Nouvel Hôpital Civil, CHRU de StrasbourgStrasbourg, France

**Keywords:** mitochondria, transmural, energetic gradient, nitric oxide, oxidative stress, heart

## Abstract

**Objective:** Left ventricle (LV) transmural gradient in mitochondrial respiration has been recently reported. However, to date, the physiological mechanisms involved in the lower endocardium mitochondrial respiration chain capacity still remain to be determined. Since, nitric oxide (NO) synthase expression in the heart has spatial heterogeneity and might impair mitochondrial function, we investigated a potential association between LV transmural NO and mitochondrial function gradient.

**Methods:** Maximal oxidative capacity (V_Max_) and relative contributions of the respiratory chain complexes II, III, IV (V_Succ_) and IV (V_TMPD_), mitochondrial content (citrate synthase activity), coupling, NO (electron paramagnetic resonance), and reactive oxygen species (ROS) production (H_2_O_2_ and dihydroethidium (DHE) staining) were determined in rat sub-endocardium (Endo) and sub-epicardium (Epi). Further, the effect of a direct NO donor (MAHMA NONOate) on maximal mitochondrial respiratory rates (V_max_) was determined.

**Results:** Mitochondrial respiratory chain activities were reduced in the Endo compared with the Epi (−16.92%; *P* = 0.04 for V_max_ and –18.73%; *P* = 0.02, for V_succ_, respectively). NO production was two-fold higher in the Endo compared with the Epi (*P* = 0.002) and interestingly, increasing NO concentration reduced V_max_. Mitochondrial H_2_O_2_ and LV ROS productions were significantly increased in Endo compared to Epi, citrate synthase activity and mitochondrial coupling being similar in the two layers.

**Conclusions:** LV mitochondrial respiration transmural gradient is likely related to NO and possibly ROS increased production in the sub-endocardium.

## Introduction

The mammalian heart presents both structural and functional heterogeneities across the left ventricular (LV) wall. Blood flow, structure, metabolism, electrophysiological, and contractile properties differ between the LV sub-endocardium (Endo) and sub-epicardium (Epi), generally supporting increased mechanical stress, contractility, and susceptibility to ischemia in Endo (Van der Vusse et al., 1990; Cazorla et al., 2005; Buckberg et al., 2008; Lou et al., 2011; Haynes et al., 2014).

Mitochondria play a critical role in ischemia/reperfusion injury and are involved both as target and source of oxidative damage (Gustafsson and Gottlieb, 2008; Lejay et al., 2014). These cell organelles produce 95% of the energy that is required by cardiomyocytes to maintain permanent contractility/relaxation cycles, participate in redox cell signaling and cell apoptosis/necrosis and are largely involved in cardiovascular diseases (Murphy et al., 2016). It is well-known that some differences exist between Endo and Epi regarding cellular properties and therefore, mitochondrial functionnal heterogeneities across the LV might also be expected. These data support the need to document the anatomical source of myocardial samples (Endo or Epi) when performing studies (Haynes and Campbell, 2014). Accordingly, we recently reported that Endo mitochondrial respiratory chain complex activities were reduced compared to Epi in normal rat left ventricle. Further, LV hypertrophy has been shown to modulate such gradient which might be a new marker of transition between complicated and uncomplicated LV hypertrophy (Kindo et al., 2012).

However, to date, the mechanisms involved in the transmural energetic gradient of the normal LV still remain to be determined. Key factors that deserve to be studied are reactive oxygen species (ROS) and nitric oxide (NO) productions. Indeed, high oxidative stress has been related to mitochondrial dysfunction both in skeletal and in cardiac muscles (Thaveau et al., 2007; Gustafsson and Gottlieb, 2008; Charles et al., 2011; Murphy et al., 2016; Paradis et al., 2016). However, unlike during cardiac hypertrophy, our previous report did not show a transmural ROS gradient in normal LV (Kindo et al., 2012).

Interestingly, NO also modulates mitochondrial respiratory chain (MRC) activity and mitochondrial permeability transition pore (mPTP) function which opening leads to the apoptotic cascade (Brown and Borutaite, 2007; Ziolo et al., 2008; Halestrap, 2009). NO is produced by the enzyme nitric oxide synthase (NOS), which catalyzes the conversion of L-arginine to L-citrulline and NO (Brown and Borutaite, 2007). Further, NO signaling pathway plays a crucial role in the pathophysiology of ischemia/reperfusion injury (Schulz et al., 2004) and studies have shown that NOS is compartmentalized within the cardiomyocytes and potentially throughout the left ventricular wall (Brahmajothi and Campbell, 1999; Barouch et al., 2002). Increased NOS and potentially ROS production in the Endo -as compared to the Epi- might therefore participate in a reduced mitochondrial respiratory chain activity in the LV endocardium.

The objectives of this study were to determine mitochondrial respiratory chain complexes activities in the normal LV and to investigate whether NO and ROS productions are specifically increased in Endo, supporting their participation in LV transmural respiratory gradient.

## Methods

### Animal care

This study conformed to the *Guide for the Care and Use of Laboratory Animals* published by the US National Institutes of Health (NIH Publication No. 85-23, revised 1996). The protocol was approved by the University of Strasbourg Institutional Animal Care and Use Committee. Twenty-three male Wistar rats (370–470 g) were used for the experiments. The rats were provided with standard food and water ad libitum.

### Rat heart harvesting

Anesthesia was first induced by 4% isoflurane gas and then maintained by reducing the isoflurane concentration to 2%. Heparin was administered intraperitoneally (10,000 UI/L) to prevent any thrombi formation in the heart and coronary arteries.

A sternotomy was performed. The still-beating heart was rapidly harvested and rinsed in ice-cold 0.9% NaCl solution, and the heart and the LV were weighed. The LV-free wall was dissected, and Endo and Epi myocardial samples were extracted under binocular microscopy (Endo and Epi were the innermost and outermost layers of the LV-free wall respectively). Tissue samples were used immediately for the measurement of mitochondrial functions and H_2_O_2_ production.

### Mitochondrial respiratory chain activity and coupling

The MRC activity was studied in saponin-skinned fibers to ensure that the global function was assessed in intact mitochondria, in their normal intracellular milieu, preserving essential interactions with other organelles (Kindo et al., 2012). Briefly, the fibers were separated and permeabilized in solution S containing saponin (50 μg/mL) for 30 min at 4°C with shaking. The permeabilized fibers were subsequently washed for 10 min with shaking to remove the saponin. The fibers were then placed in a bath with the respiratory solution for 5 min twice to remove any phosphates. Finally, oxygen consumption was measured polarographically with a Clark-type electrode in a 3 ml oxygraphic cell (Strathkelvin Instruments, Glasgow, Scotland). The basal oxygen consumption (V_0_) and maximal fiber respiration (V_max_) rates were measured at 22.1°C with continuous stirring in the presence of a saturating concentration of adenosine diphosphate (ADP) as a phosphate acceptor.

The relative contribution of respiratory chain complexes I, III, and IV to the global mitochondrial respiratory rate was also determined using different mitochondrial substrates and inhibitors (Thaveau et al., 2007). When V_max_ was recorded, the electron flow through complexes I, III, and IV, because both glutamate (5 mM) and malate (2 mM) were present. Complex I was inhibited with amytal (0.02 mM), and complex II was stimulated with succinate (25 mM). Mitochondrial respiration under these conditions allowed us to determine the activity of complexes II, III and IV (V_succ_). Subsequently, N, N, N′, N′-tetramethyl-p-phenylenediamine dihydrochloride (TMPD, 0.5 mM), and ascorbate (0.5 mM) were added as artificial electron donors for cytochrome c. Under these conditions, cytochrome c oxidase (complex IV) activity was maximal, and this was defined as an isolated step in the respiratory chain (V_tmpd_). After the experiments, fibers were harvested and dried for 15 min at 150°C and respiration rates were expressed as μmol O2/min/g dry weight.

Mitochondrial coupling is inferred from the acceptor control ratio (V_max_/V_0_) which indicates the degree of coupling between oxidation and phosphorylation. V_max_ and V_0_ represent the maximal and the basal mitochondrial respiratory rates, respectively.

### Nitric oxide (NO) production

Small LV tissue samples (3.0−5.0 mg dry fiber weight) were minced and placed into separate wells of a 24-well plate with 2 mL KHB (99 mM NaCl, 4.69 mM KCl, 2.5 mM CaCl_2_, 1.2 mM MgSO_4_, 25 mM NaHCO_3_, 1.03 mM KH_2_PO_4_, 5.6 mM D-(+)-glucose, and 20 mM Na-HEPES, pH 7.4) that contained 200 μM FeSO_4_ and 415 μM DETC, which were previously degassed with N_2_. The tissue pieces were then incubated at 37°C with 20 mmHg O_2_ for 1 h and then placed on ice.

LV tissue preparation was performed as previously described (Fink et al., 2006). The tissue pieces were frozen in a syringe containing 1 ml KHB in liquid nitrogen immediately after incubation, and the samples were stored at −80°C prior to EPR analysis. Quantification of the Fe (DETC)^2−^ nytrosyl concentration in the EPR tissue was performed at −180°C in a frozen dewar (Noxygen Science Transfer and Diagnostics, Elsach, Germany). The EPR settings were as follows: center field *g* = 2.03, field sweep = 80 G, microwave power = 43.60 mW, modulation amplitude = 8.80 G, conversion time = 20.48 ms and time constant = 81.92 ms. NO production is expressed as mg dry fiber weight/minute. The NO concentration in the LV tissue was calculated from a standard curve relating the NO concentration to the EPR signal amplitude.

### Effect of NO on mitochondrial respiratory chain activity

To investigate the effect of NO on MRC activity, the maximal fiber respiration (V_max_) rate was also measured in the presence of increasing concentrations of the NO donor MAHMA NONOate (10, 40, 60, and 80 μM) with a saturating amount of ADP as a phosphate acceptor. MAHMA NONOate is spontaneously dissociated with a half-life of 3 min at 22−25°C and liberates 2 moles of NO per mole of parent compound.

The fibers were then harvested and dried for 15 min at 150°C. The respiration rates are expressed as μmol O_2_/min/g dry weight.

### Hydrogen peroxide production in permeabilized fibers

The H_2_O_2_ production in response to the sequential addition of substrates and inhibitors was assessed in permeabilized Endo and Epi fibers (15). H_2_O_2_ production was measured with Amplex Red reagent (Invitrogen); this reagent reacts in 1:1 stoichiometry with H_2_O_2_ in a reaction catalyzed by HRP (horseradish peroxidase; Fluka Biochemika) to yield the fluorescent compound resorufin and a molar equivalent O_2_. Resorufin has excitation/emission wavelengths of 563/587 nm and is highly stable once formed. Fluorescence was measured continuously [change in fluorescence (F)/s] with a Fluoromax 4 (Jobin Yvon) spectrofluorometer equipped with temperature control and magnetic stirring.

After the baseline in the presence of F (reactants only) was established, the reaction was initiated by addition of a permeabilized fiber bundle to 600 μl of buffer Z with glutamate (5 mM) and malate (2.5 mM) as substrates for complex I and succinate (5 mM) as a substrate for complex II. The addition of ADP (2 mM) to the reaction buffer led to a reduction in H_2_O_2_ release, as expected when the electron flow through the respiratory chain is stimulated. Finally, addition of the complex I inhibitor amytal (2 mM) and the complex III inhibitor antimycin (8 μM) led to interruption of the normal electron flow and induced an increase in the H_2_O_2_ release. At the conclusion of each experiment, fibers were harvested and dried for 15 min at 150°C. H2O2 production was expressed as pmol/min/mg dry weight.

### Dihydroethidium staining

To evaluate *in situ* production of superoxide in the Endo and the Epi, serial sections (10 μm thick) were cut with a cryostat microtome and mounted onto glass slides. The slides were air-dried and incubated for 30 min at 37°C with 2.5 μM dihydroethidium (DHE) in phosphate-buffered saline (PBS). DHE produces a red fluorescence when oxidized to ethidium bromide by the superoxide anion. After staining, the sections were washed, air-dried, mounted in a Vectashield® (Vector Laboratories Inc., Burlingame, CA) and covered with a cover slip. The slides were examined with an epifluorescence microscope (Nikon Eclipse E800) and a 20X epifluorescence objective; the emission signal was recorded with a Zeiss 573−637 nm filter.

### Citrate synthase activity

We evaluated the global mitochondrial content in Endo and Epi layers by measuring the activity of citrate synthase (Kindo et al., 2012). Citrate synthase activity was expressed as units of activity per gram of tissue wet weight (IU.gww).

### Mitochondrial permeability transition pore (mPTP) opening

Permeabilized fibers without myosin were prepared as described previously (Halestrap, 2009). Briefly, ghost fibers were prepared by incubating saponin-permeabilized bundles for 30 min with agitation at 4°C in a high-KCl buffer, which allows myosin to be extracted. Ca^2+^ accumulation in the mitochondrial matrix is the most important trigger for mPTP opening. mPTP opening was assessed as described previously (Picard et al., 2008).

Briefly, we measured the mitochondrial Ca^2+^ uptake after the addition of a single Ca^2+^ pulse (20 nM). After the pulse was applied, we monitored the decrease in the extramitochondrial calcium concentration with the fluorescent probe Calcium Green-5N (Invitrogen). The progressive uptake of Ca^2+^ by the mitochondria was monitored until mitochondrial Ca^2+^ release caused by mPTP opening was observed. The mitochondrial calcium retention capacity (CRC), which is a reliable index of mPTP sensitivity, was calculated as the total amount of Ca^2+^ taken up by mitochondria before Ca^2+^ release. The CRC values are expressed as mg dry fiber weight (Pottecher et al., 2016).

### Statistical analysis

The values are expressed as the mean ± standard error of mean (SEM). Differences in the group means were statistically analyzed by the Mann Whitney test or a one-way analysis of variance (ANOVA) with the Newman-Keuls post-test analysis. A *P* < 0.05 was considered to be statistically significant. Statistical analysis was performed with the Prism software (GraphPad Prism 5, Graph Pad Software Inc., San Diego, CA, USA).

## Results

### Mitochondrial content and coupling, and transmural differences in mitochondrial respiration across the normal left ventricle: reduced subendocardial mitochondrial respiratory chain complex activities

**Figure 1:**
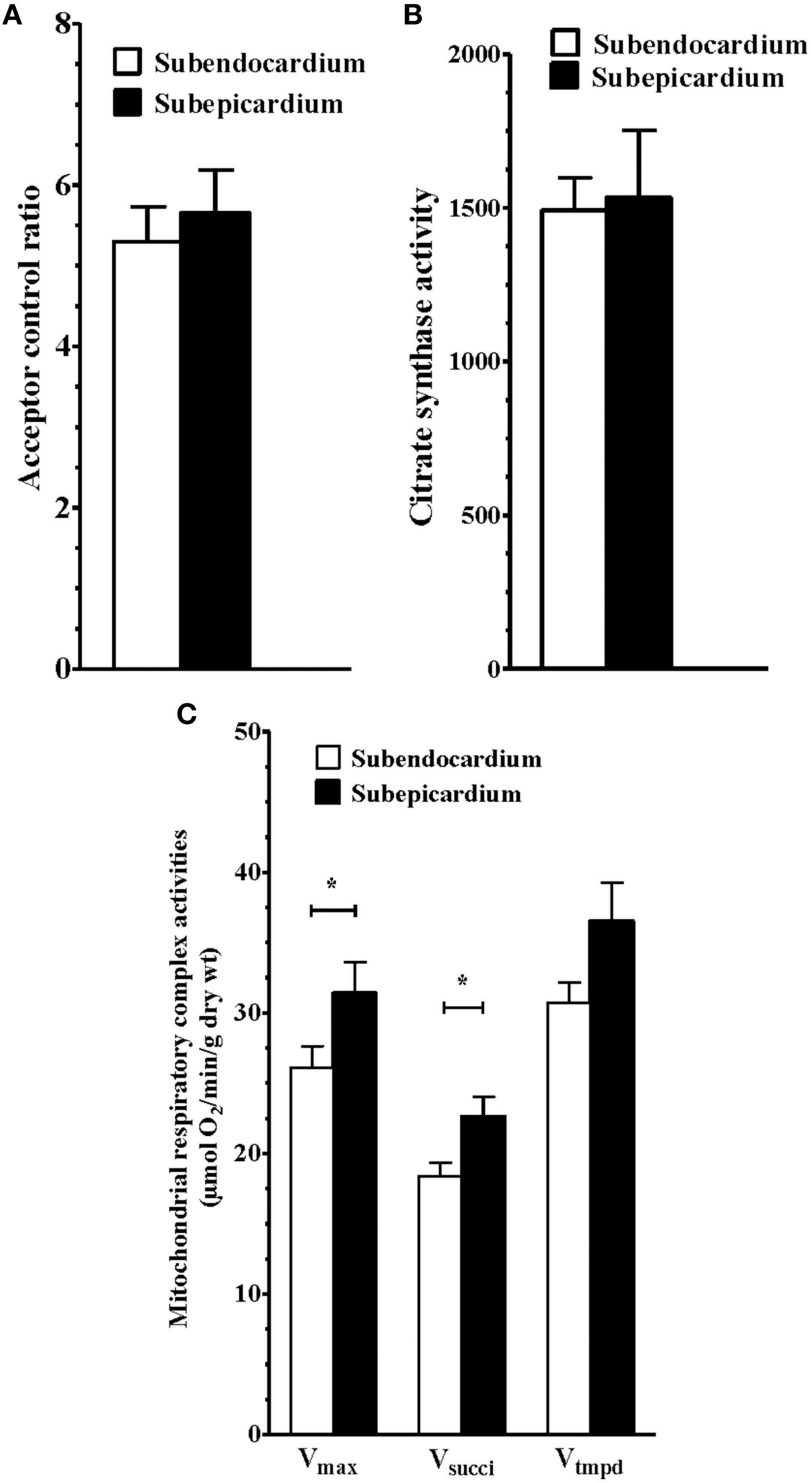
**Similar coupling and mitochondrial content but reduced sub-endocardium respiratory chain complex activities**. **(A)** Subendocardium and subepicardium mitochondrial coupling. **(B)** Subendocardium and subepicardium citrate synthase activity. **(C)** Subendocardium and subepicardium mitochondrial respiratory chain complex activities. V_max_, complexes I, III, and IV activities; V_succi_, complexes II, III, and IV activities; V_tmpd_, complex IV activity. Values are means ± SEM. ^*^P < 0.05.

No difference through the LV wall was found regarding the global mitochondrial content by measuring the activity of citrate synthase (Figure 1A; 1510 ± 80.3 vs. 1430 ± 103.5 mUI/min/g dry weight, Endo vs. Epi respectively, *P* not significant).

Similarly, the acceptor control ratio (V_max_/V_0_) indicating the degree of coupling between oxidation and phosphorylation, was similar in the two myocardial layers (Figure 1B; 5.43 ± 0.44 vs. 5.94 ± 0.47 for the Endo and Epi, respectively; *P* >0.05).

However, maximal mitochondrial respiratory rates (V_max_, complexes I, III, and IV) were significantly reduced in the Endo compared with the Epi (Figure 1C; 26.07 ± 1.54 vs. 31.38 ± 2.20 μmol O2/min/g dry weight, respectively; −16.92%; *P* = 0.040, *n* = 13).

Likewise, there was a consistent decrease in complex II, III, and IV activity in the presence of succinate substrate (V_succi_) in the Endo (Figure 1B; 18.39 ± 0.94 vs. 22.63 ± 1.39 μmol O2/min/g dry weight, respectively; −18.73%; *P* = 0.025).

No difference between the Endo and Epi respiratory activity was observed with TMPD-ascorbate substrates (complex IV) (Table 1).

**Table 1:** **A comparison of the subendocardial and subepicardial MRC with various substrates and inhibitors**.

	**Myocardial fibers**		***P-value* Subepicardium vs Subendocardium**.
	**Subepicardium**	**Subendocardium**	
V_0_	5.80 ± 0.74	5.15 ± 0.48	0.738
V_max_	31.38 ± 2.20	26.07 ± 1.54	**0.040**
V_succi_	22.63 ± 1.39	18.39 ± 0.94	**0.025**
V_tmpd_	36.50 ± 2.77	30.70 ± 1.45	0.260

*ADP (V_max_), succinate (V_succi_), TMPD-ascorbate (V_tmpd_)*.

*The data are presented as the mean ± SEM*.

*V_o_, basal oxygen consumption; V_max_, complexes I, III, and IV activities; V_succi_, complexes II, III, and IV activities; V_tmpd_, complex IV activity*.

*The two values have been transcribed in bold to assess the statistical significance of these values (P < 0.05)*.

Thus, normal LV is characterized by transmural differences in mitochondrial respiratory chain complexes I, II, and III activities.

### Transmural differences in nitric oxide production across the normal left ventricle and effects of NO on mitochondrial respiratory chain complex activities

#### Increased subendocardial NO production

**Figure 2:**
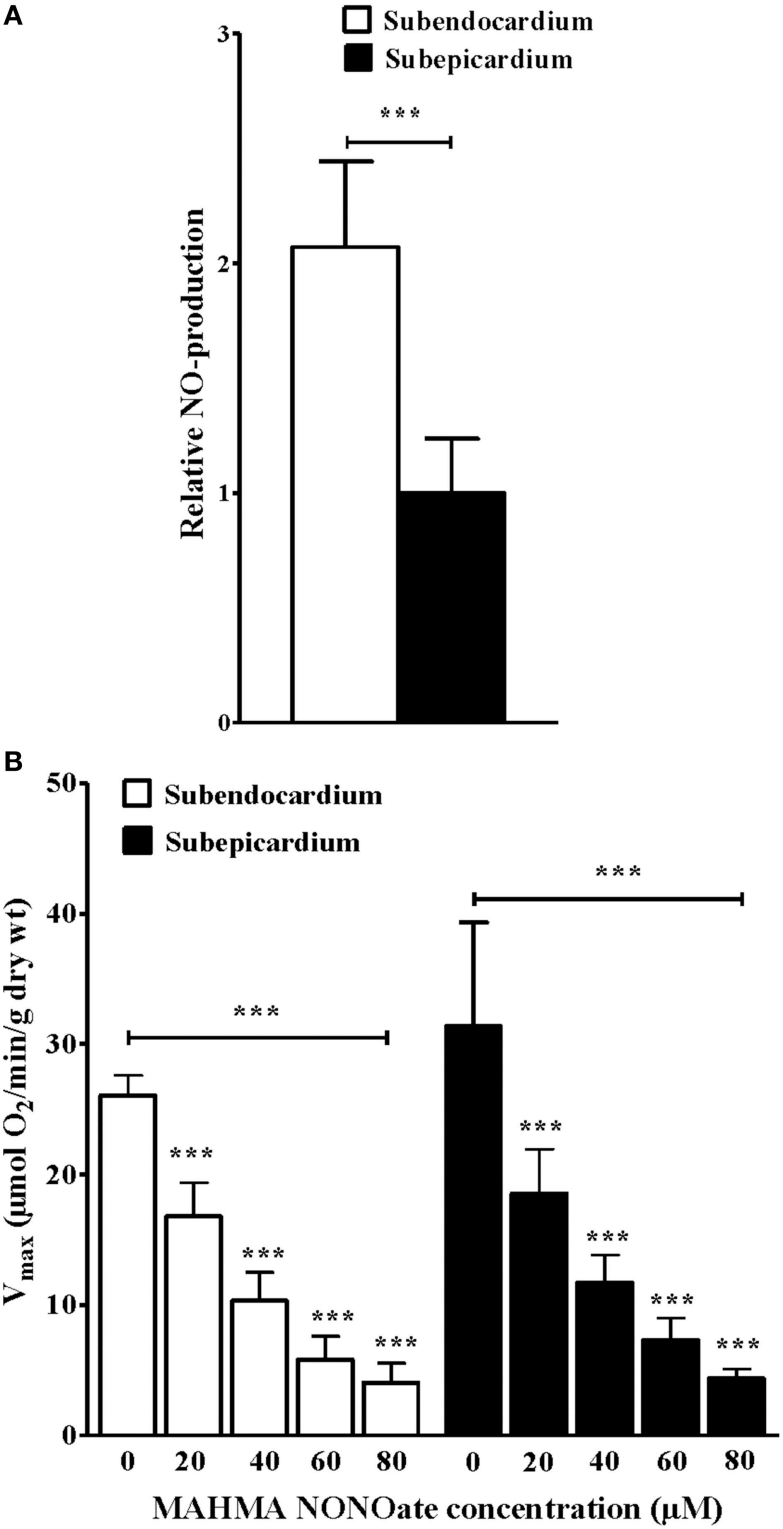
**Increased subendocardium nitric oxide production and deleterious effects of increasing NO donor concentrations on mitochondrial oxidative capacity**. **(A)** NO detection in subendo- and in subepi-cardium using electron paramagnetic resonance with the Fe(DETC)_2_ colloid. **(B)** V_max_ with increasing concentrations of the NO donor MHMA NONOate in permeabilized ENDO and EPI fibers. The data are presented as the mean ± SEM. ^***^P < 0.001.

NO production was assessed using EPR (*n* = 10 rats), which indicated that the NO level was increased approximately two-fold in the Endo compared with the Epi (Figure 2A; 1.00 ± 0.23 vs. 2.07 ± 0.37 relative NO-production, respectively; *P* = 0.002).

#### NO impairs subendocardial and subepicardial mitochondrial respiratory chain complex activities

With increasing concentrations of MAHMA NONOate, which is a NO donor, the maximal respiratory rates were significantly decreased in the Endo and the Epi (Figure 2B; *n* = 10 rats). There was a dose-dependent response of V_max_ to the NO donor. At a concentration of 40 μM MAHMA NONOate, the subendocardial V_max_ was decreased 65.6% and the subepicardial V_max_ was decreased 61.0% compared with the basal V_max_ (Figure 2B).

### Transmural differences in reactive oxygen species production across the normal left ventricle: increased subendocardial H_2_O_2_ production and DHE staining

**Figure 3:**
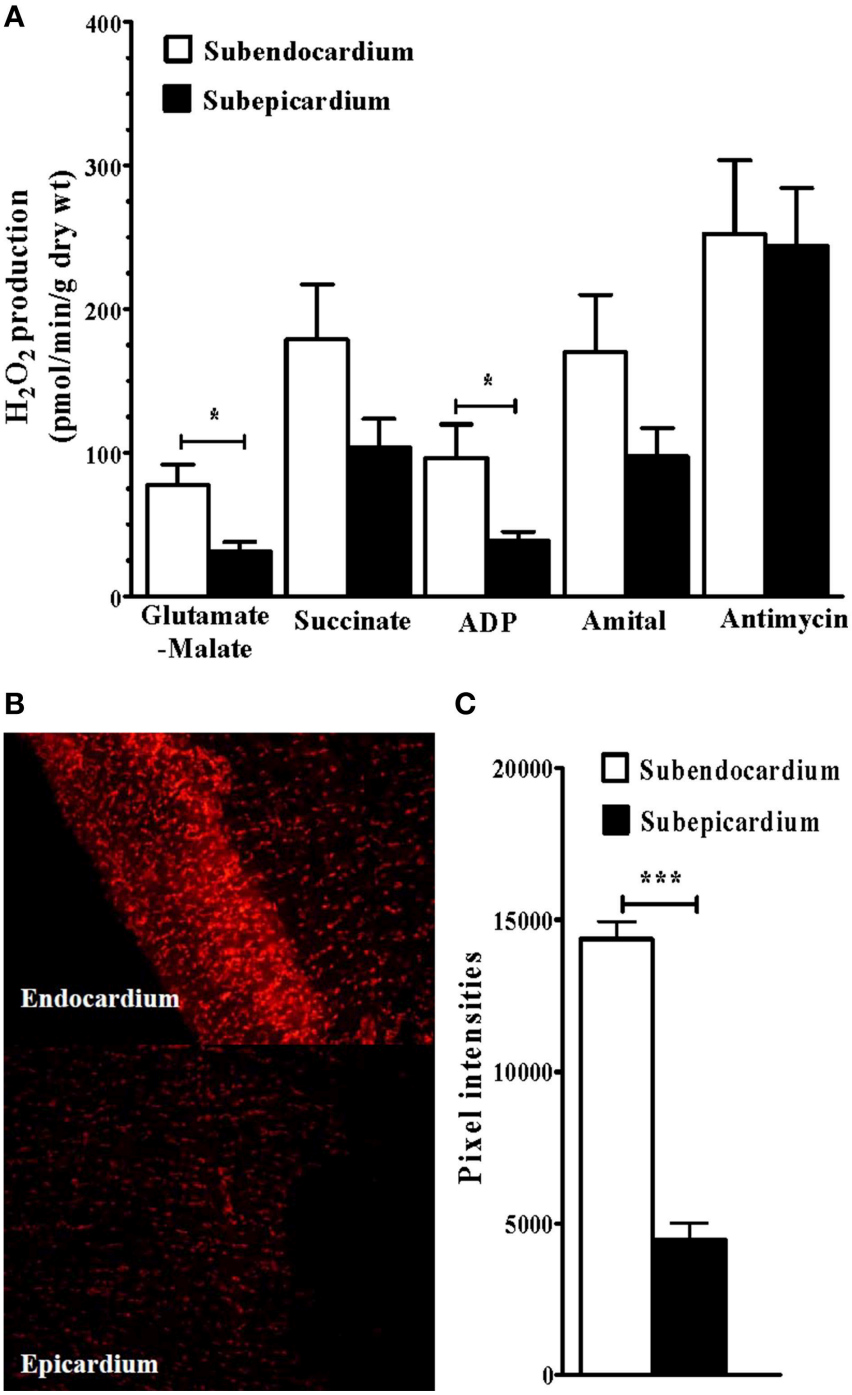
**Increased subendocardium reactive oxygen species production**. **(A)** Mitochondrial H_2_O_2_ production in subendo- and in subepicardium. **(B)** Examples of dihydroethidium (DHE) staining. **(C)** Dihydroethidium (DHE) fluorescence intensity (arbitrary units). Glutamate-Malate, complex I substrates. Succi: succinate, complex II substrate. ADP: adenosine diphosphate, ATP synthase substrate. Amytal, complex I inhibitor. Antimycin A, complex III inhibitor. Values are means ± SEM. ^*^P < 0.05; ^***^P < 0.001.

Mitochondrial H_2_O_2_ production was significantly increased in the Endo compared with the Epi (*n* = 12 rats; Figures 3A). In the presence of complex I glutamate-malate donors, H_2_O_2_ release was significantly increased in the Endo compared with the Epi (77.54 ± 14.20 vs. 31.40 ± 6.59 pmol/min/mg dry weight, respectively; +59.50%; *P* = 0.025). When the complex II donor succinate was added, no statistical difference was observed between the 2 myocardial layers. In the presence of the ADP substrate, H_2_O_2_ release was increased in the Endo compared with the Epi (96.59 ± 23.19 vs. 38.75 ± 6.22 pmol/min/mg dry weight, respectively; +59.88%; *P* = 0.030). These results suggest that complexes I and III of the MRC are responsible for H_2_O_2_ production.

Moreover, superoxide anion production, which was measured with DHE fluorescence intensity, was significantly increased in the Endo (Figures 3B,C; *n* = 10).

### Mitochondrial permeability transition pore opening is similar in Endo and Epi layers

**Figure 4:**
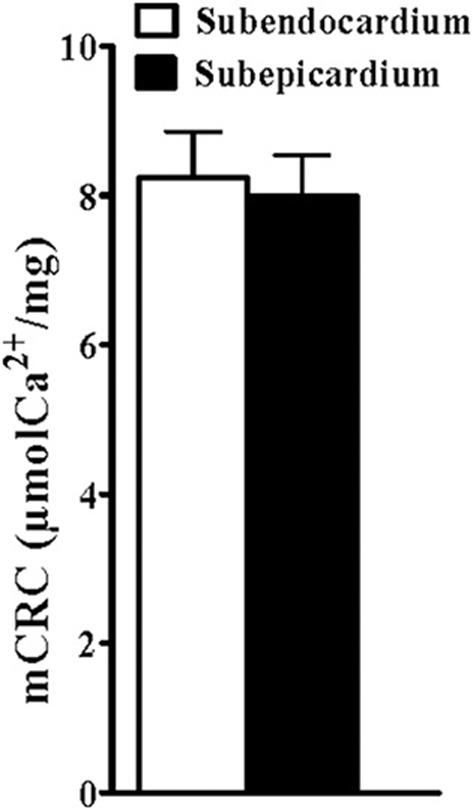
**Similar mitochondrial calcium retention capacity**. mCRC: mean calcium retention capacity corresponding to the quantity of calcium load needed to open the mitochondrial permeability transition pore as assessed using Calcium Green fluorometry. Values are means ± SEM.

The sensitivity of mPTP opening was assessed fluorometrically using Calcium Green (13 rats). There was no statistical difference between the mitochondrial Ca^2+^ retention capacity for the Endo and the Epi (Figure 4).

## Discussion

The main results of this study are to confirm a lower oxidative capacity of the sub-endocardium as compared to the sub-epicardium across the normal left ventricle, together with an increased nitric oxide concentration in the endocardium. Reactive oxygen species production was also enhanced in LV endocardium. Mitochondrial content and coupling are similar in both LV layers.

### Reduced subendocardial mitochondrial respiratory chain complex activities

Despite accumulating evidences regarding myocardial heterogeneity of the normal heart, upon blood flow, structure, metabolism, electrophysiological, and contractile properties, there are few data investigating potential energetic gradient across the LV wall.

Particularly, there are only two reports using saponin-skinned fibers that ensure global mitochondrial functionnal assessment in intact mitochondria (i.e., not isolated and potentially fragilized mitochondria). MacDonald et al. observed no LV transmural respirational difference in healthy rats (MacDonald et al., 2011). On the other hand, we recently observed reduced mitochondrial respiration in the Endo as compared to the Epi (Kindo et al., 2012). Such a discrepancy was not explained since both animal models and mitochondrial respiration investigations were similar.

In the present study, subendocardial oxidative capacity was significantly lower than subepicardial capacity when using glutamate/malate or succinate, supporting that complexes I, II, and III activities of the mitochondrial respiratory chain were reduced. This result gave further *corpus* to the existence of a transmural energetic gradient in the LV wall and suggests the need to investigate potential mechanisms involved. Since, as previously reported (Kindo et al., 2012), mitochondrial content is similar in both layers, two main candidates, NO and ROS deserve to be studied, both potentially inhibiting the mitochondrial respiration rate.

### Increased subendocardial NO production

NO is a free radical with a very short live in tissue. Under physiological conditions, NO detection and quantification in biological tissue is challenging (Hogg, 2010). Actually EPR NO spin-trapping allows to assess the NO tissue concentration under physiological conditions with high sensibility and specificity (Hogg, 2010).

EPR spin-trapping showed that NO level was significantly higher in the LV Endo compared with the LV Epi. Our data highlight for the first time, to our knowledge, that a transmural NO gradient exists through the LV at the basal state. Accordingly, Brahmajothi et al. have shown heterogeneous expression gradients of NOS in the LV wall with NOS1 predominately localized in LV Endo and NOS3 in LV Epi (Brahmajothi and Campbell, 1999). Taken together, these findings suggest that the compartmentalization of NOS expression is likely associated with a transmural NO gradient through the LV wall.

The physiological significance of such transmural NO gradient is still poorly known but increased NO concentration in the subendocardium may participate in the higher contractility and blood flow of the Endo compared with the Epi, previously observed in mammalian hearts (Brahmajothi and Campbell, 1999; Moore et al., 2000). Indeed, there is evidence that NOS1 has a positive inotropic effect in the heart and NO might enhance blood flow, helping maintain an adequate O_2_ concentration in the Endo (Ziolo et al., 2008).

Further, some new informations might be inferred from our results. Indeed, NO increase in the Endo might have been linked with the reduced subendocardial mitochondrial respiratory chain complex activities. Thus, despite a similar global mitochondrial content through the LV wall, the NO gradient observed in this study was associated with reduced complexes I, II, and III activities in the Endo compared with the Epi. To further analyze a potential relationship between increased NO and decreased mitochondrial respiratory chain function, we determined the effect of increasing dose of NO donor on the maximal oxidative capacity of both Endo and Epi LV layers. V_max_ activities decreased when the NO donor was added. This is consistent with previous data demonstrating that NO can inhibit mitochondrial respiration by reversible direct inhibition of cytochrome c oxidase and by reactive nitrate species production leading to respiratory complexes S-nitrosylation (Brown and Borutaite, 2007; Lima et al., 2010). Further, exercise-induced cardioprotection might prevent excessive NO synthesis and therefore peroxynitrite formation (Farah et al., 2013).

### Increased subendocardial ROS production

ROS include free radicals like superoxide anion, hydroxyl and the highly reactive compound hydrogen peroxide (H_2_O_2_). H_2_O_2,_ resulting from superoxide dismutase action on superoxide anion, can give rise to hydroxyls radicals and may impair mitochondrial respiration rates (Veal et al., 2007). Accordingly, high oxidative stress has been related to mitochondrial dysfunction during ischemia-reperfusion and therapies that decreased DHE staining were associated with mitochondrial function normalization (Charles et al., 2011).

In the present study, H_2_O_2_ overproduction and increased DHE staining were observed in Endo, supporting an increased oxidative stress in the sub-endocardium as compared to the sub-epicardium. Such increased oxidative stress, specifically located in the Endo, might have been involved in sub-endocardium reduced mitochondrial function. However, alternatively, mitochondrial dysfunction is also a source of ROS production and mitochondrial reduced respiration might have been the origin of H_2_O_2_ overproduction and increased DHE staining (reflecting ROS production including superoxide anion, Pottecher et al., 2013) in this study.

Interestingly, however a reduced Endo mitochondrial function was observed despite similar relative H_2_O_2_production in both Endo and Epi layers (Kindo et al., 2012). Thus, taken together, although the relationship between ROS production and mitochondrial oxidative capacity in Endo remains to be further studied, ROS appears not necessary in LV transmural energetic gradient genesis.

### Similar mitochondrial permeability transition pore sensitivity in both Endo and Epi layers

To investigate whether reduced sub-endocardial mitochondrial respiratory chain complex activities and/or whether increased sub-endocardial NO production might be considered as deleterious, we determined for the first time the mitochondrial permeability transition pore sensitivity in both Endo and Epi layers. Although mitochondrial membrane permeabilization can occur independent of mPTP formation, mPTP is a major large ion channel in the inner mitochondrial membrane that induces apoptosis upon opening (Halestrap, 2009). mPTP sensitivity to Ca^2+^ was constant throughout the left ventricular wall further supporting the physiological context of our study, performed in a normal heart.

In summary, our data give an overview of left ventricular wall physiology at the basal state. A transmural NO gradient, present in normal left ventricle might potentially participate in the endocardium higher contractility and blood flow. However, increased subendocardial NO concentration is associated with a decrease in the mitochondrial respiratory chain complex activity and with an increase in mitochondrial ROS production.

Such associations should probably not be viewed as impairments since they are observed in normal hearts and since the sensitivity of mPTP opening, involved in apoptosis, is similar in left ventricular wall layers.

Further studies will be useful to demonstrate if a defect in this transmural heterogeneity in NO expression modulates mitochondrial function and participate in the pathophysiology of cardiovascular diseases. Indeed, we previously observed that the level of the transmural gradient in mitochondrial respiration might be used as potential biomarker for transition from uncomplicated to complicated LV hypertrophy.

## Author contributions

Conception or design of the work: MK, SG, JB, TH, AC, FP, BG. Acquisition, analysis: MK, SG, JB, TH, AC, JZ. Interpretation of data for the work: MK, SG, JM, JZ, FP, BG. Drafting or revising the work: MK, AC, JM, BG. Final approval: MK, SG, JB, TH, AC, JM, JZ, FP, BG. Agreement to be accountable of all aspects of the work: MK, SG, JB, TH, AC, JM, JZ, FP, BG.

## Funding

This work was supported by a grant from the OCOVAS (‘Association des opérés du coeur et des vaisseaux à Strasbourg’) and the RITAC (‘Recherche et Innovations Technologiques dans les Affections Cardiovasculaires’).

### Conflict of interest statement

The authors declare that the research was conducted in the absence of any commercial or financial relationships that could be construed as a potential conflict of interest. The reviewer AS and handling Editor declared their shared affiliation, and the handling Editor states that the process nevertheless met the standards of a fair and objective review.
